# Inpatient Hospitalization Costs Associated with Birth Defects Among Persons Aged <65 Years — United States, 2019

**DOI:** 10.15585/mmwr.mm7227a1

**Published:** 2023-07-07

**Authors:** Justin Swanson, Elizabeth C. Ailes, Janet D. Cragan, Scott D. Grosse, Jean Paul Tanner, Russell S. Kirby, Norman J. Waitzman, Jennita Reefhuis, Jason L. Salemi

**Affiliations:** ^1^Lawton and Rhea Chiles Center for Healthy Mothers and Babies, College of Public Health, University of South Florida, Tampa, Florida; ^2^Division of Birth Defects and Infant Disorders, National Center on Birth Defects and Developmental Disabilities, CDC; ^3^Office of the Director, National Center on Birth Defects and Developmental Disabilities, CDC; ^4^Department of Economics, University of Utah, Salt Lake City, Utah.

**Summary**

**What is already known about this topic?**

Estimates of birth defect–associated hospitalization costs must be updated as detection, diagnosis, and treatment evolve for numerous birth defects.

**What is added by this report?**

During 2019, among patients aged <65 years, 4.1% of all hospitalizations and 7.7% of related inpatient medical costs were associated with birth defects. The total estimated cost of birth defect–associated hospitalizations was $22.2 billion.

**What are the implications for public health practice?**

These updated estimates of hospitalization costs illustrate the importance of continually determining the health care needs of persons with birth defects to ensure optimal health for all.

## Abstract

Changing treatments and medical costs necessitate updates to hospitalization cost estimates for birth defects. The 2019 National Inpatient Sample was used to estimate the service delivery costs of hospitalizations among patients aged <65 years for whom one or more birth defects were documented as discharge diagnoses. In 2019, the estimated cost of these birth defect–associated hospitalizations in the United States was $22.2 billion. Birth defect–associated hospitalizations bore disproportionately high costs, constituting 4.1% of all hospitalizations among persons aged <65 years and 7.7% of related inpatient medical costs. Updating estimates of hospitalization costs provides information about health care resource use associated with birth defects and the financial impact of birth defects across the life span and illustrates the need to determine the continued health care needs of persons born with birth defects to ensure optimal health for all.

## Introduction

In the United States, major structural birth defects attributable to genetic, chromosomal, teratogenic, or unknown etiologies affect approximately 3% of live births (*1*) and are the leading cause of infant mortality, responsible for 21% of newborn and infant deaths (*2*). Their treatments incur significant financial costs throughout a person’s lifetime. As treatments and medical costs change, updates to hospitalization cost estimates for birth defects are needed.

## Methods

Developed for the Healthcare Cost and Utilization Project (HCUP), the National Inpatient Sample (NIS) is the largest publicly available, all-payor inpatient care database in the United States.* NIS uses a 20% systematic sampling of all discharges from short-term, nonfederal community hospitals. Because NIS does not identify patients across multiple hospital visits, the unit of analysis for this study is individual hospitalization rather than individual patient. To reduce the impact of potential miscoding of age-related abnormalities as birth defects (particularly cardiovascular defects) (*3*), only patients aged <65 years discharged during January 1–December 31, 2019 were included. Birth hospitalizations were determined separately from other hospitalizations during the first year of life to better differentiate the costs of birth defects from routine delivery costs. Records missing values of age or billed charges were excluded. Sampling weights for the remaining hospitalizations were adjusted to retain total hospitalization frequency and cost.

Cost estimates were calculated as the product of the amount billed for a hospitalization and the corresponding hospital-level cost-to-charge ratios. HCUP-provided cost-to-charge ratios are computed on an annual basis for each hospital (total institutional service delivery costs divided by total amount charged by the hospital).^†^ NIS records facility charges but not professional fees charged by physicians who are not hospital employees.

Birth defects were identified by scanning up to 40 available diagnosis code fields associated with each hospitalization among codes Q00–Q99 (congenital malformations, deformations, and chromosomal abnormalities) of the *International Classification of Diseases, Tenth Revision, Clinical Modification* (ICD-10-CM). Patent ductus arteriosus (Q25.0) and atrial septal defect (Q21.1) were not considered birth defects when occurring in neonates aged <28 days or with an associated indicator of preterm birth (P07.2 or P07.3). In addition, 23 conditions classified within the Q00–Q99 code range that are commonly considered benign or are otherwise unlikely to contribute to hospitalization costs were not considered birth defects for the purposes of this analysis (Supplementary Table, https://stacks.cdc.gov/view/cdc/130207).

Sampling weights were applied to calculate national estimates of hospitalization frequency and cost. Mean, median, and total costs were calculated by patient demographic and birth defect code characteristics. Mean hospitalization costs were stratified by age group for selected individual birth defects and birth defect categories. If a hospitalization was associated with more than one defect included in the table, the full cost of the hospitalization was included for each defect. The individual birth defects listed include those defined in the National Birth Defects Prevention Network Congenital Malformations Surveillance Report.^§^ Statistical analyses were performed using SAS software (version 9.4; SAS Institute) with survey procedures incorporating sampling design. This activity was reviewed by CDC and was conducted consistent with applicable federal law and CDC policy.^¶^

## Results

During 2019, a total of 937,295 birth defect–associated hospitalizations incurred a total cost of $22,204,754,855 (Table 1), representing 4.1% of hospitalizations and 7.7% of hospitalization costs among persons in the United States aged <65 years. A birth defect code was the principal diagnosis code for 15.8% of all birth defect–associated hospitalizations that were not birth hospitalizations.

**TABLE 1:** Weighted national estimates of frequencies and costs of hospitalizations with at least one birth defect–associated discharge diagnosis* among persons aged <65 years, by selected characteristics — National Inpatient Sample, United States, 2019

Characteristic	No. of discharges (%)	Total cost, USD (%)	Mean cost, USD	Median cost, USD
**All hospitalizations with a birth defect diagnosis**	**937,295 (100.0)**	**22,204,754,855 (100.0)**	**23,690**	**7,054**
**Position of birth defect diagnosis code, excluding birth hospitalizations (N = 583,665)**				
Principal diagnosis	92,200 (15.8)	**4,628,169,299 (26.7)**	50,197	21,837
Not principal diagnosis	491,465 (84.2)	**12,734,760,311 (73.3)**	25,912	10,693
**Age group at admission, yrs**				
Birth	353,630 (37.7)	**4,841,825,245 (21.8)**	13,692	1,668
<1, nonbirth	97,085 (10.4)	**6,007,670,516 (27.1)**	61,881	15,708
1–5	70,405 (7.5)	**2,010,690,220 (9.1)**	28,559	11,495
6–18	84,330 (9.0)	**2,406,631,722 (10.8)**	28,538	13,745
19–64	331,845 (35.4)	**6,937,937,152 (31.2)**	20,907	11,050
**Co-occurring preterm birth diagnosis code, first year of life only (N = 450,715)**				
Yes	69,145 (15.3)	**4,982,419,450 (45.9)**	72,058	23,567
No	381,570 (84.7)	**5,867,076,311 (54.1)**	15,376	1,865
**Primary payer**				
Medicare/Medicaid	489,435 (52.2)	**11,830,918,831 (53.3)**	24,173	7,228
Private insurance	377,260 (40.2)	**8,624,077,051 (38.8)**	22,860	6,892
Self-pay/No charge	38,420 (4.1)	**589,451,660 (2.7)**	15,342	4,454
Other	30,810 (3.3)	**1,131,579,789 (5.1)**	36,728	10,309
Missing	1,370 (0.1)	**28,727,525 (0.1)**	20,969	8,138
**Race or ethnicity**				
Black or African American, non-Hispanic	155,790 (16.6)	**3,643,653,444 (16.4)**	23,388	5,453
White, non-Hispanic	465,705 (49.7)	**10,623,669,704 (47.8)**	22,812	8,254
Hispanic or Latino, all races	159,090 (17.0)	**4,176,577,475 (18.8)**	22,752	6,770
Other race, non-Hispanic	99,905 (10.7)	**2,468,402,379 (11.1)**	26,253	5,128
Missing	56,805 (6.1)	**1,292,451,853 (5.8)**	24,707	3,550
**Birth defects category^†^**				
Cardiovascular	209,045 (22.3)	**9,833,000,308 (44.3)**	47,038	15,750
Cardiovascular, critical	32,380 (3.5)	**2,810,676,170 (12.7)**	86,803	29,430
Central nervous system	97,810 (10.4)	**3,170,662,573 (14.3)**	32,417	11,920
Chromosomal	81,450 (8.7)	**2,629,174,588 (11.8)**	32,280	10,393
Cleft lip or palate or both	13,450 (1.4)	**371,907,278 (1.7)**	27,651	9,457
Ear	16,505 (1.8)	**311,185,562 (1.4)**	18,854	1,979
Eye	7,020 (0.7)	**353,579,767 (1.6)**	50,367	13,268
Gastrointestinal	58,630 (6.3)	**2,372,323,342 (10.7)**	40,463	12,043
Genitourinary	190,550 (20.3)	**3,827,722,973 (17.2)**	20,088	5,702
Integumentary	185,165 (19.7)	**1,968,999,140 (8.9)**	10,634	1,587
Musculoskeletal	152,150 (16.2)	**4,139,059,463 (18.6)**	27,204	8,948
Other syndrome affecting multiple systems	29,050 (3.1)	**1,040,175,603 (4.7)**	35,806	12,161
Other defect	42,875 (4.6)	**2,757,425,472 (12.4)**	64,313	11,987

**Abbreviations:** ICD-10-CM = *International Classification of Diseases, Tenth Revision, Clinical Modification*; USD = U.S. dollars.

* Identified by scanning up to 40 available fields for ICD-10-CM diagnosis codes Q00–Q99: congenital malformations, deformations, and chromosomal abnormalities. Patent ductus arteriosus (Q25.0) and atrial septal defect (Q21.1) were not considered birth defects when occurring in neonates aged <28 days or with an associated indicator of preterm birth (ICD-10-CM codes P07.2 or P07.3). An additional 23 conditions of lesser clinical importance classified within the Q00–Q99 code range were not considered birth defects.

^†^ Birth defects categories correspond to blocks of ICD-10-CM diagnosis codes: cardiovascular (Q20–Q28), central nervous system (Q00–Q07), chromosomal (Q90–Q99), cleft lip/palate (Q35–Q37), ear (Q16–Q17), eye (Q10–Q15), gastrointestinal (Q38–Q45), genitourinary (Q50–Q64), integumentary (Q80–Q85), musculoskeletal (Q65–Q79), and other syndrome (Q87). “Other defect” includes birth defects of the face and neck (Q18), respiratory system (Q30–Q34), and other congenital malformations (Q86 and Q89). Critical cardiovascular defects (https://www.cdc.gov/ncbddd/heartdefects/facts.html) were operationalized using the National Birth Defects Prevention Network Congenital Malformations Surveillance Report (https://www.nbdpn.org/docs/Birth_Defects_Data_and_Directory_2022.pdf). The specific defects included are only those defined in the National Birth Defects Prevention Network Congenital Malformations Surveillance Report; birth defects not included contribute to frequencies and costs aggregated by birth defect category and overall. Because a single hospitalization might include more than one specific birth defect, individual birth defect frequencies might sum to >100% of birth defects categories or overall frequencies, and mean costs multiplied by frequencies will correspondingly sum to >100% of total costs.

Nonbirth hospitalizations of persons aged <1 year at admission were associated with the highest mean ($61,881) and median ($15,708) costs per hospitalization among all age groups (Table 1). Among birth defect–associated hospitalizations during the first year of life, a co-occurring preterm birth diagnosis code was present in 15.3% of hospitalizations, and hospitalizations with a preterm birth diagnosis code were associated with 45.9% of hospitalization costs.

The most prevalent birth defect category was cardiovascular defects (22.3%), which were associated with a total cost of $9,833,000,308 (44.3% of all birth defect–associated hospitalization costs) (Table 1). Critical cardiovascular defects alone accounted for 12.7% of birth defect–associated hospitalization costs and had the highest median cost ($29,430) per hospitalization. Among nonbirth hospitalizations of neonates and infants, defects with a mean cost >$150,000 included esophageal atresia ($214,651), interrupted aortic arch ($199,973), and diaphragmatic hernia ($195,456) (Table 2). Although mean costs per nonbirth hospitalization were highest for patients aged <1 year, this was not consistent across individual birth defects (Table 2) (Figure). Overall, nearly one third of hospitalization costs occurred among patients aged 19–64 years (Table 1). 

**TABLE 2:** Weighted national estimates of frequencies and mean costs of birth defect–associated hospitalizations* among persons aged <65 years, by selected specific birth defect and age group — National Inpatient Sample, United States, 2019

Birth defect^†^	Age group at admission, yrs											
	Birth hospitalization		<1, nonbirth		1–5		6–18		19–64		All age groups	
	No.^§^	Mean cost, USD	No.^§^	Mean cost, USD	No.^§^	Mean cost, USD	No.^§^	Mean cost, USD	No.^§^	Mean cost, USD	No.^§^	Mean cost, USD
**No defect**	3,571,404	4,804	416,265	25,979	398,395	14,516	951,630	14,670	16,658,885	13,099	**21,996,579**	**12,089**
**Any defect**	353,630	13,692^¶^	97,085	61,881	70,405	28,559	84,330	28,538	331,845	20,907	**937,295**	**23,690**
**Cardiovascular, critical**	5,965	79,481	11,440	128,700	5,380	79,335	3,770	63,332	5,825	34,105	**32,380**	**86,803**
**Cardiovascular**	40,095	46,527	41,690	85,846	21,490	45,754	15,720	44,526	90,050	30,043	**209,045**	**47,038**
Aortic valve stenosis	340	73,405	490	119,278	110	27,810	280	61,876	1,370	41,825	**2,590**	**62,196**
Atrial septal defect	—**	—**	15,680	63,964	9,490	42,483	5,095	35,458	45,570	31,479	**75,835**	**39,840**
Atrioventricular septal defect	1,180	104,798	2,820	117,756	1,030	60,301	515	52,148	520	27,665	**6,065**	**92,182**
Coarctation of aorta	1,720	71,865	2,525	135,489	615	46,094	570	45,512	1,680	31,249	**7,110**	**80,521**
Common truncus	185	141,256	205	130,074	130	161,151	155	66,602	135	50,004	**810**	**112,124**
Dextro-transposition of great arteries	855	83,149	1,295	170,533	480	79,142	310	55,015	660	37,539	**3,600**	**103,264**
Double outlet right ventricle	805	111,159	1,640	127,862	1,110	77,471	490	78,043	315	77,532	**4,360**	**102,714**
Ebstein anomaly	170	77,141	285	95,984	300	46,389	225	64,109	750	27,301	**1,730**	**51,611**
Hypoplastic left heart syndrome	915	121,117	2,075	150,444	1,470	73,969	945	92,979	380	74,707	**5,785**	**112,011**
Interrupted aortic arch	115	102,776	320	199,973	95	77,770	60	69,036	25	10,323	**615**	**142,438**
Pulmonary valve atresia	200	272,866	380	124,374	190	173,504	150	41,734	150	45,257	**1,070**	**138,177**
Single ventricle	340	155,126	1,010	166,406	750	66,991	485	61,920	515	42,193	**3,100**	**104,135**
Tetralogy of Fallot	1,365	73,218	3,050	117,165	920	126,979	735	52,117	1,210	29,541	**7,280**	**89,034**
Total anomalous pulmonary venous connection	270	106,042	760	189,531	230	55,991	115	65,213	75	21,751	**1,450**	**134,265**
Tricuspid valve atresia	300	92,262	660	81,529	430	83,806	205	53,565	440	24,579	**2,035**	**68,462**
Ventricular septal defect	19,100	35,719	12,555	77,434	4,415	38,583	2,025	39,583	4,900	26,875	**42,995**	**47,369**
**CNS**	13,865	52,329	11,600	71,978	14,400	24,901	19,005	28,716	38,940	18,127	**97,810**	**32,417**
Anencephaly	255	3,502	—**	—**	15	49,903	15	78,230	—**	—**	**285**	**9,877**
Encephalocele	265	33,206	315	64,542	120	24,526	205	66,508	1,745	25,496	**2,650**	**34,037**
Holoprosencephaly	330	48,100	430	52,217	615	22,643	550	34,333	175	21,945	**2,100**	**35,703**
Spina bifida without anencephaly	880	45,169	1,250	64,401	1,675	18,846	4,675	25,621	21,140	15,491	**29,620**	**20,225**
**Chromosomal**	8,210	46,993	10,995	75,070	15,225	26,372	15,235	28,578	31,785	18,281	**81,450**	**32,280**
Trisomy 13	265	35,177	285	63,489	180	24,873	200	48,340	170	36,940	**1,100**	**43,492**
Trisomy 18	585	56,072	520	106,796	450	37,366	230	31,700	405	18,974	**2,190**	**54,852**
Trisomy 21	4,700	37,462	6,225	59,353	6,740	20,740	5,425	23,676	19,955	16,716	**43,045**	**26,654**
Turner syndrome	455	20,837	185	66,991	210	37,083	435	25,589	2,360	17,867	**3,645**	**22,760**
**Cleft lip, palate, or both**	4,595	20,809	4,205	44,472	2,225	20,661	1,640	17,551	785	18,513	**13,450**	**27,651**
Cleft lip with cleft palate	1,965	23,733	1,805	49,534	795	17,313	1,000	15,135	340	20,581	**5,905**	**29,118**
Cleft lip without cleft palate	915	5,896	450	15,407	50	10,883	60	13,972	80	8,569	**1,555**	**9,258**
Cleft palate without cleft lip	1,715	25,414	1,950	46,493	1,380	22,944	580	22,086	365	18,765	**5,990**	**30,980**
**Ear**	13,050	10,005	1,715	77,822	710	31,595	715	26,105	315	19,229	**16,505**	**18,854**
Anotia or microtia	680	18,657	360	99,759	305	33,708	575	26,824	110	26,292	**2,030**	**38,028**
**Eye**	2,085	64,266	1,605	82,823	1,165	28,803	825	35,048	1,340	18,047	**7,020**	**50,367**
Anophthalmia or microphthalmia	255	68,830	345	66,729	210	20,661	130	34,154	160	16,837	**1,100**	**47,315**
Congenital cataract	235	24,404	190	61,187	160	41,027	135	22,791	265	11,517	**985**	**30,511**
**GI**	11,040	55,032	16,210	62,397	5,525	34,895	4,460	29,743	21,395	19,998	**58,630**	**40,463**
Biliary atresia	665	154,509	1,355	100,377	340	74,293	210	38,767	340	34,661	**2,910**	**97,576**
Esophageal atresia or tracheoesophageal fistula	845	66,710	920	214,651	255	51,169	110	47,589	790	26,491	**2,920**	**100,363**
Rectal and large intestinal atresia or stenosis	1,310	57,601	2,415	65,145	860	21,769	345	20,196	370	16,827	**5,300**	**49,943**
Small intestinal atresia or stenosis	1,100	88,157	1,055	151,120	325	31,938	145	24,159	185	17,017	**2,810**	**97,308**
**Genitourinary**	80,320	14,354	15,555	68,294	8,385	26,362	8,450	22,150	77,840	15,472	**190,550**	**20,088**
Congenital posterior urethral valves	220	55,495	565	73,936	325	28,558	365	32,728	130	42,875	**1,605**	**50,332**
Hypospadias	13,330	13,411	2,150	60,831	685	23,908	280	24,946	1,385	21,013	**17,830**	**20,304**
Renal agenesis or hypoplasia	2,150	23,577	1,185	64,522	1,155	45,675	1,680	22,996	13,370	17,399	**19,540**	**23,089**
**Integumentary**	158,620	6,788	5,760	79,162	2,310	22,121	3,505	26,692	14,970	19,479	**185,165**	**10,634**
**MS**	57,260	18,657	18,095	74,183	13,845	27,660	20,460	27,166	42,490	18,584	**152,150**	**27,204**
Clubfoot	8,215	14,914	1,810	89,692	1,315	20,961	1,445	20,056	2,100	16,226	**14,885**	**25,225**
Craniosynostosis	960	30,115	3,035	47,606	1,485	34,028	680	36,084	205	19,316	**6,365**	**39,658**
Diaphragmatic hernia	1,040	110,848	915	195,456	390	22,978	155	40,803	755	23,732	**3,255**	**100,562**
Gastroschisis	1,325	85,080	505	186,873	155	18,598	150	14,763	200	12,744	**2,335**	**91,969**
Limb deficiencies or reduction defects	1,125	21,628	350	70,995	495	31,571	655	26,979	790	18,491	**3,415**	**28,430**
Omphalocele	980	48,074	460	158,502	335	40,173	75	32,640	105	10,330	**1,955**	**70,084**
**Other syndrome affecting multiple systems**	1,725	66,503	3,555	92,066	4,560	31,502	5,065	28,560	14,145	21,905	**29,050**	**35,806**
**Other defect**	9,645	107,240	8,035	138,524	7,165	34,298	6,310	23,981	11,720	18,173	**42,875**	**64,313**

**Abbreviations:** CNS = central nervous system; GI = gastrointestinal; ICD-10-CM = *International Classification of Diseases, Tenth Revision, Clinical Modification*; MS = musculoskeletal; USD = U.S. dollars.

* Identified by scanning up to 40 available diagnosis code fields for ICD-10-CM diagnosis codes Q00–Q99: congenital malformations, deformations, and chromosomal abnormalities.

^†^ Birth defects categories correspond to blocks of ICD-10-CM diagnosis codes: cardiovascular (Q20–Q28), CNS (Q00–Q07), chromosomal (Q90–Q99), cleft lip/palate (Q35–Q37), ear (Q16–Q17), eye (Q10–Q15), GI (Q38–Q45), genitourinary (Q50–Q64), integumentary (Q80–Q85), MS (Q65–Q79), and other syndrome (Q87). “Other defect” includes birth defects of the face and neck (Q18), respiratory system (Q30–Q34), and other congenital malformations (Q86 and Q89). Critical cardiovascular defects (https://www.cdc.gov/ncbddd/heartdefects/facts.html) were operationalized using the National Birth Defects Prevention Network Congenital Malformations Surveillance Report (https://www.nbdpn.org/docs/Birth_Defects_Data_and_Directory_2022.pdf). The specific defects included are only those defined in the National Birth Defects Prevention Network Congenital Malformations Surveillance Report; birth defects not included here contribute to frequencies and costs aggregated by birth defect category and overall.

^§^ Because a single hospitalization might include more than one specific birth defect, individual birth defect frequencies might sum to >100% of birth defects categories or overall frequencies, and mean costs multiplied by frequencies will correspondingly sum to >100% of total costs.

^¶^ For hospitalizations with any birth defect, the birth hospitalization costs include the costs for routine birth hospitalization.

** Cell sizes ≤10 were suppressed. Sums across all ages exclude suppressed cells.

**FIGURE:**
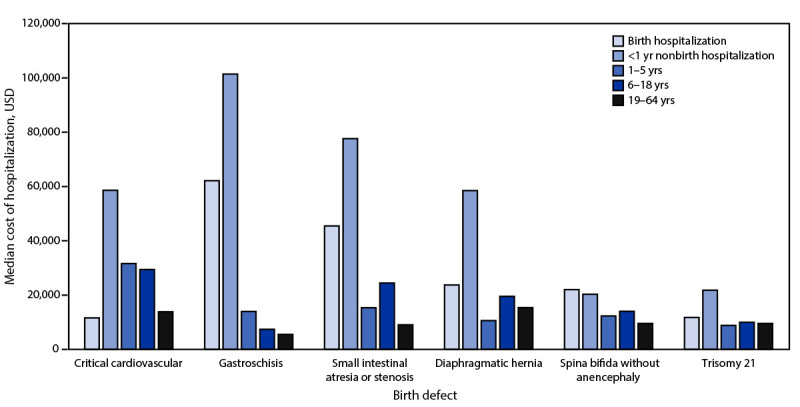
Weighted estimates of median costs of hospitalizations, by birth defect*^,^† and age group at admission — National Inpatient Sample, United States, 2019 **Abbreviation:** USD = U.S. dollars. * Identified by scanning up to 40 available fields of *International Classification of Diseases, Tenth Revision, Clinical Modification* diagnosis codes. Critical cardiovascular defects include common arterial trunk (Q20.0), double outlet right ventricle (Q20.1), transposition of the great arteries (Q20.3), single ventricle (Q20.4), tetralogy of Fallot (Q21.3), pulmonary valve atresia (Q22.0), tricuspid valve atresia (Q22.4), Ebstein anomaly (Q22.5), hypoplastic left heart syndrome (Q23.4), coarctation of aorta (Q25.1), interrupted aortic arch (Q25.21), and total anomalous pulmonary venous connection (Q26.2). Specific birth defects are identified as gastroschisis (Q79.3), small intestinal atresia or stenosis (Q41), diaphragmatic hernia (Q79.0 and Q79.1), spina bifida without anencephaly (Q05, Q07.01, and Q07.03), and trisomy 21 (Q90). ^† ^The specific birth defects shown were selected to represent a range of body systems.

## Discussion

During 2019, the cost of hospitalizations for persons aged <65 years with a birth defect diagnosis code was estimated at $22.2 billion. These hospitalizations were associated with disproportionately high costs, constituting 4.1% of all hospitalizations among persons in the United States aged <65 years and 7.7% of total costs. Nearly one half of hospitalization costs associated with birth defects occurred among neonates and infants, and these costs disproportionately affected persons aged <1 year during nonbirth hospitalizations. Defects of the cardiovascular system were the most prevalent birth defects, were associated with disproportionately high ($9.8 billion) hospitalization costs, and included many of the costliest individual birth defects.

Using 2013 NIS data, the total cost of birth defect–associated hospitalizations was estimated to be $22.9 billion for all ages and $19.1 billion for persons aged <65 years (*4*). Those estimates included adjustments for professional fees, which historically added 20%–25% to facility costs (*5*). Applying the same adjustments to the current estimates yields a 2019 estimate of $26.6–27.8 billion in total birth defect–associated hospitalization costs for persons aged <65 years. When adjusted to 2019 hospital care prices, the 2013 cost estimate is $21.0 billion for persons aged <65 years.** The share of birth defect–associated expenditures among total hospitalization expenditures was similar among all age groups: 5.2% in 2013 and 5.5% in 2019.

### Limitations

The findings in this report are subject to at least five limitations. First, determining which costs are directly attributable to birth defects is challenging because of difficulties in identifying all sequelae of birth defects and their respective codes, the coding of minor birth defects, and the possible miscoding of some acquired structural or functional abnormalities as birth defects (*3*). Excluding hospitalizations for persons aged ≥65 years reduces the risk for miscoding but fails to identify the contribution of birth defects among this age group. Second, the potential for two or more birth defects to be documented during the same hospitalization could lead to overestimation of costs for findings presented by individual defect or category. Third, cost-to-charge ratios calculated at the hospital level do not necessarily accurately reflect the costs of different types of hospital services (*6*), which could bias estimates of a person’s hospitalization costs in an unknown direction. Fourth, HCUP costs are limited to facility fees and fail to include physician or professional fees, thereby underestimating birth defect–associated hospitalization costs. Finally, it is difficult to distinguish the relationship between preterm birth and birth defects, and some of the birth defect–associated costs among preterm infants were possibly due to their prematurity rather than their birth defect.

### Implications for Public Health Practice

Updated estimates of hospitalization costs for specific birth defects provide critical information about health care resource use. These data highlight the financial impact across the life span and illustrate the need to understand the continued health care needs of persons born with birth defects to ensure optimal health for all.

## Footnotes

*
https://www.hcup-us.ahrq.gov/db/nation/nis/NISIntroduction2019.pdf

†
https://www.hcup-us.ahrq.gov/reports/methods/MS2021-05-CCR-Methodologies.pdf

§
https://www.nbdpn.org/docs/Birth_Defects_Data_and_Directory_2022.pdf

¶ 45 C.F.R. part 46; 21 C.F.R. part 56; 42 U.S.C. Sect. 241(d), 5 U.S.C. Sect. 552a, 44 U.S.C. Sect. 3501 et seq.

**
https://meps.ahrq.gov/about_meps/Price_Index.shtml
